# Phase space representation of neutron monitor count rate and atmospheric electric field in relation to solar activity in cycles 21 and 22

**DOI:** 10.1186/s40623-016-0504-3

**Published:** 2016-07-15

**Authors:** H. G. Silva, I. Lopes

**Affiliations:** 1 Renewable Energies Chair, Institute of Earth Sciences, IIFA, University of Évora, Palácio do Vimioso, Largo Marquês de Marialva, Apartado 94, 7002-554 Évora, Portugal; 2 Centro Multidisciplinar de Astrofísica, Instituto Superior Técnico, Universidade de Lisboa, Av. Rovisco Pais, 1049-001 Lisbon, Portugal

**Keywords:** Solar cycle activity, Neutron monitor count rate, Potential gradient, Space–earth weather

## Abstract

Heliospheric modulation of galactic cosmic rays links solar cycle activity with neutron monitor count rate on earth. A less direct relation holds between neutron monitor count rate and atmospheric electric field because different atmospheric processes, including fluctuations in the ionosphere, are involved. Although a full quantitative model is still lacking, this link is supported by solid statistical evidence. Thus, a connection between the solar cycle activity and atmospheric electric field is expected. To gain a deeper insight into these relations, sunspot area (NOAA, USA), neutron monitor count rate (Climax, Colorado, USA), and atmospheric electric field (Lisbon, Portugal) are presented here in a phase space representation. The period considered covers two solar cycles (21, 22) and extends from 1978 to 1990. Two solar maxima were observed in this dataset, one in 1979 and another in 1989, as well as one solar minimum in 1986. Two main observations of the present study were: (1) similar short-term topological features of the phase space representations of the three variables, (2) a long-term phase space radius synchronization between the solar cycle activity, neutron monitor count rate, and potential gradient (confirmed by absolute correlation values above ~0.8). Finally, the methodology proposed here can be used for obtaining the relations between other atmospheric parameters (e.g., solar radiation) and solar cycle activity.

## Introduction

Atmospheric electricity measurements are of crucial importance in the study of space– and earth–weather relations (Harrison et al. 2013). In these measurements, the variability in the thunderstorm activity serves as one of its most significant verifications (Velinov et al. 1992; Scott et al. 2014; Owens et al. 2014). A physical mechanism explaining the influence of the solar cycle activity on the thunderstorm activity was initially proposed by Markson (1981), based on the observation of a positive correlation between galactic cosmic rays (GCRs) and the ionospheric potential (*V*_I_). This potential generates a vertical conduction current between the ionosphere and Earth’s surface, forming the so-called global electric circuit (GEC) (Tonev 2011; Velinov and Tonev 2008; Conceição and Silva 2015). Moreover, GCRs are the primary cause of Earth’s electrification, and higher GCR levels tend to decrease the surface potential gradient (PG^1^) by increasing the atmospheric electric conductivity (Nicoll and Harrison 2014; Mateev and Velinov 1992). Based on this mechanism, PG measurements are expected to be negatively correlated with GCRs. In fact, comparison between the neutron monitor count rate (NC^2^) in Climax, Colorado (USA), and PG measurements in Lisbon (Portugal) confirmed such a relation (Serrano et al. 2006), but no further relation between the PG and solar cycle activity was sought. Nevertheless, a link between the solar cycle activity and PG is likely to exist, because GCRs are severely modulated by the solar activity (Potgieter 2013). Note that GCRs are considered to be composed of anti-protons, electrons, positrons, and ionized nuclei that are not produced inside the heliosphere. They exhibit clear 11- and 22-year-long cycles owing to the solar modulation (Usoskin et al. 2009). Such modulation is mainly controlled by the transport of GCRs under the influence of the field lines of the solar magnetic field, as explained by Thomas et al. (2013). Stronger solar magnetic activity implies stronger magnetic fields, weakening the transport of GCRs. The inverse appears to hold for a weaker solar activity. As a consequence, the solar cycle activity is negatively correlated with GCRs, as shown by Potgieter (2013). This implies, in turn, that the solar cycle activity is negatively correlated with the NC and should be positively correlated with the PG, based on the arguments above.

In terms of solar physics, sunspot area (SSA) and sunspot number (SSN) are often used as proxies of the solar magnetic field activity (Lopes et al. 2014). This implies that stronger solar magnetic activity corresponds to higher SSA/SSN, while weaker solar magnetic activity corresponds to lower SSA/SSN. Important insights into the solar magnetic activity were indeed gained, recently, using low-order dynamo models (LODMs) in the analysis of SSA/SSN time series (Passos and Lopes 2008a; Lopes and Passos 2009a). Nonlinear behavior of LODMs is, in fact, a well-established property of the solar dynamo, shown from first principles, validated by numerical dynamo simulations and aximetrical dynamo models (Charbonneau 2010). Another essential aspect of LODMs is the phase space representation (PSR) of the solar magnetic field, and a detailed description of this technique can be found in (Passos and Lopes 2008b) and references therein. In this representation, borrowed from the chaos theory, the time derivative of a given variable, $v(t) \equiv \dot{x}(t),$ is plotted against that same variable, *x*(*t*). This method enables to inspect the oscillatory nature of the system under study and was used for demonstrating Van der Pol–Duffing oscillator-like characteristics of the SSA/SSN time series, known now to be a fundamental property of the solar dynamo (Lopes et al. 2014; Lopes and Passos 2009b). For this reason, the PSR will be used here to investigate the relations between the SSA, NC, and PG, on short and long timescales, and to characterize long-term synchronization between these variables. In addition, models based on Van der Pol–Duffing oscillators are likely to be of interest for analyzing the NC and PG. This does not necessarily imply that the solar magnetic field and the two variables have the same underlying Physics; rather, they could exhibit similar nonlinear behavior. This may generate significant insights into coupling mechanisms among the three. Along the same line of thought, Blanter et al. (2014, 2016) used the Kuramoto model with two nonlinear oscillators coupled to reconstruct the phase evolution of the toroidal and poloidal components of the solar magnetic field. Those authors used time series of SSN and geomagnetic indices as proxies of the toroidal and poloidal components, respectively. The finding of phase synchronization between SSN and geomagnetic indices (Blanter et al. 2014) constitutes another motivation for the present PSR analysis.

## Data

Hourly PG values were registered at the Portela meteorological station (Lisbon, Portugal, 38°47′N, 9°08′W), and the measurements were made using a Benndorf electrograph. In the present study only the period from 1978 to 1990 was considered, owing to the radioactive fallout that followed the nuclear tests in the 1960s and earlier 1970s (Serrano et al. 2006; Silva et al. 2014). Data series of cosmic radiation flux are not available in Lisbon. Therefore, surface hourly NCs recorded at the Climax, Colorado, station (39°37′N, 106°18′W) were used as a measure of GCRs entering the Earth’s troposphere. This station is located at the geomagnetic latitude ~47°N, relatively close to that of Lisbon, Portugal, which is ~40°N. The main reason for using the NC data from Climax, Colorado, was because the present analysis followed previous work by Serrano et al. (2006), who used the same PG and NC data. It is important to mention that other European NC stations could be considered (e.g., Rome neutron monitor or Athens neutron monitor stations), measuring at rigidities, *R*_c_, closer to the ones of Lisbon. However, for the sake of consistency with previous work this comparison will be considered in the future work. The SSA was measured in units of millionths of the Sun’s hemisphere and made available by NOAA. The daily time series data considered here correspond to global SSA. The period studied covers two solar cycles: (1) solar cycle 21, beginning in June 1976 and ending in September 1986 (lasting 10.3 years, with maximal activity around December 1979), and (2) solar cycle 22, beginning in September 1986 and ending in May 1996 (lasting 9.7 years, with maximal activity around July 1989). Daily averages were computed for both the PG and NC time series, for consistency with the SSA. Note that the data contain two solar maxima (one in 1979 and another in 1989) and one solar minimum (in 1986); this implies that the results presented below are representative from the solar cycle activity point of view.

## Results and discussion

**Fig. 1:**
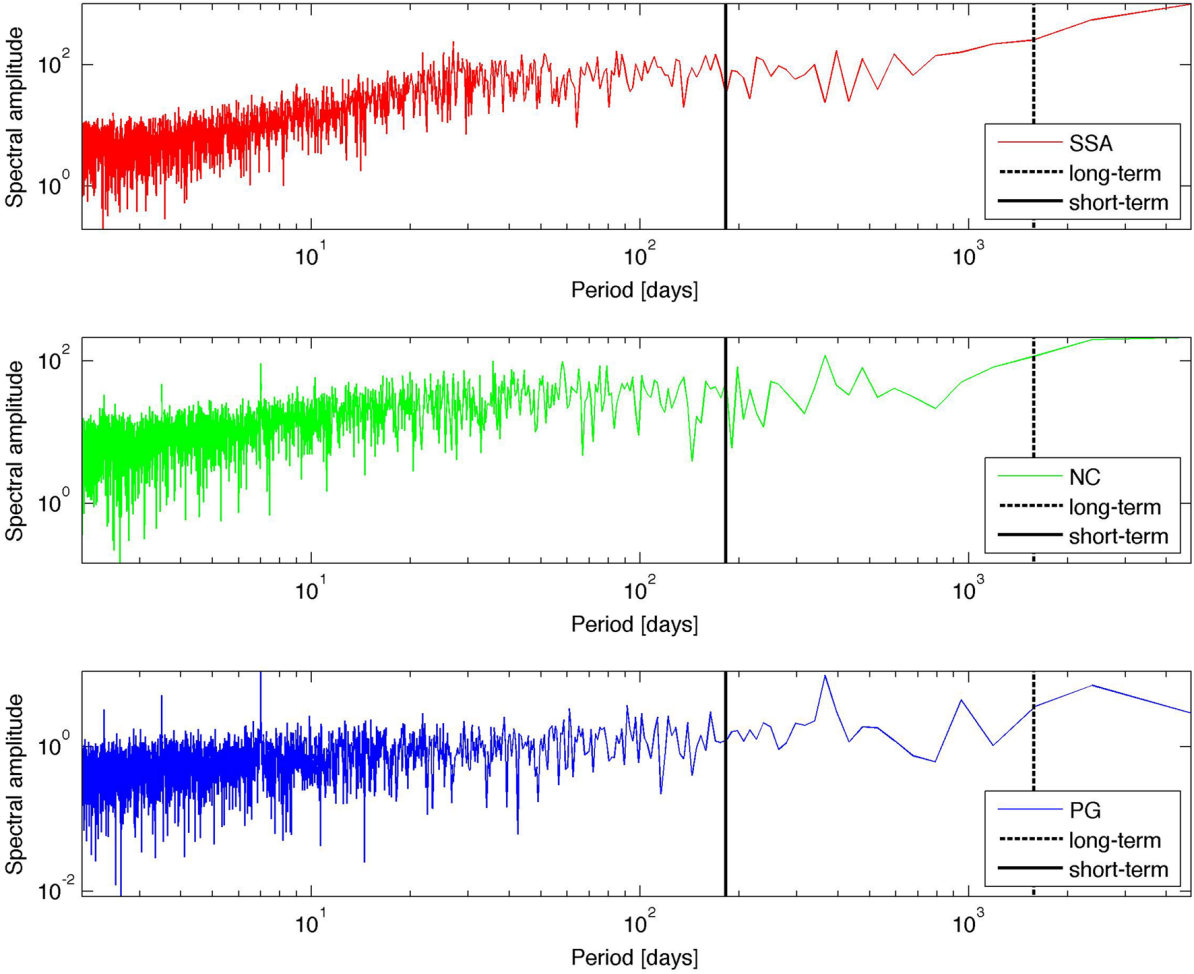
Fast Fourier transforms (FFTs) of: the sunspot area (*upper panel*), the neutron monitor count rate (*middle panel*), and the potential gradient (*lower panel*)

As noted by Lockwood (2012), the effects of the solar cycle activity are convolved with other influences on Earth’s atmosphere. For this reason, appropriate timescales need to be found. Two timescales will be considered here: (1) a short timescale (within the SSA variability) and (2) a long timescale (within the solar cycle modulation); a detailed description is provided below. Figure 1 shows the fast Fourier transform (FFT) of the time series in the periodogram representation (representing periods instead of frequencies). From this representation, smoothed data series can be reconstructed, preserving periods above a given lower period, by applying a rectangular filter to the FFT and inverting it. In this way, two signals can be generated for each variable:

### Short term (ST)

Signals with periodicities ranging from 6 months (0.5 years) (marked by the first solid vertical line in Fig. 1) to the end of the periodogram, *x*_st_. This timescale filters the 27-day-long variation in the SSA (Lopes and Silva 2015), the dominant weekly cycle (7 days) and the daily cycle (1 day) affecting the PG (Silva et al. 2014), as well as fast atmospheric processes affecting Earth’s Atmosphere, such as disturbed weather conditions. The ST timescale has been used in previous PSRs of the SSA (Lopes et al. 2014).

### Long term (LT)

Signals preserving only three longer periods, ~4.34, ~6.50, and ~13.00 years, *x*_lt_, that filter all low-periodicity contributions below ~4.34 years. The ~4.34 years period is marked by the second dashed vertical line in Fig. 1. These periods are the only three periods in the FFT that are longer than ~4.34 years. The LT timescale is usually related to the solar cycle activity (Lopes and Silva 2015).

**Fig. 2:**
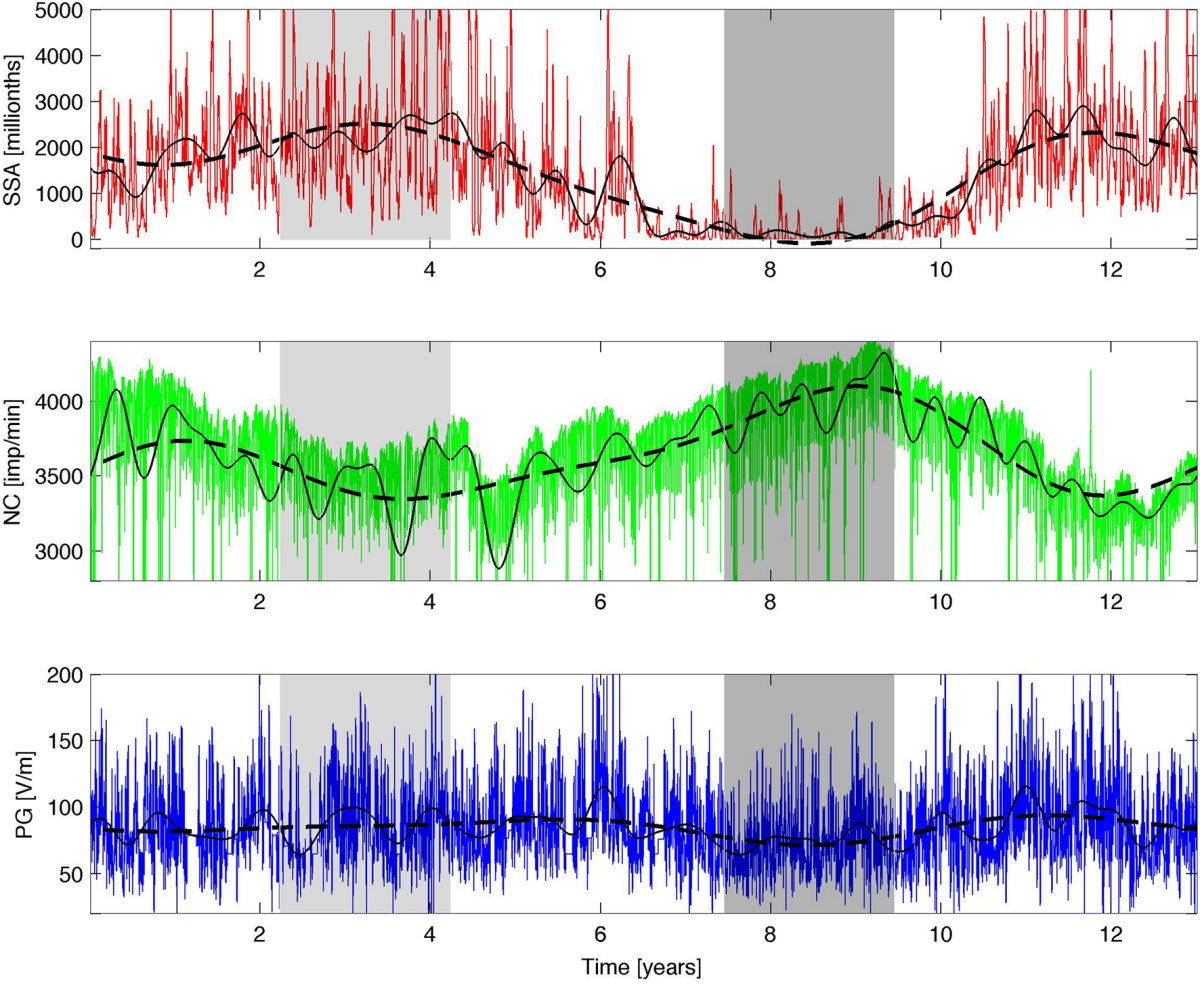
Raw data and filtered signals for: the sunspot area (*upper panel*); the neutron monitor count rate (*middle panel*); the potential gradient (*lower panel*). The *first gray*-*shaded* area corresponds to the 1979 solar maximum, while the *second gray*-*shaded area* corresponds to the 1986 solar minimum

Figure 2 shows the original signals (colored lines) and the filtered ones (solid lines for *x*_st_ and dashed lines for *x*_lt_). The first gray-shaded area in the time series corresponds to 1 year before and 1 year after the 1979 solar maximum, while the second gray-shaded area corresponds to 1 year before and 1 year after the 1986 solar minimum. Note that the inversion of the FFT preserves the spectral amplitude and angular phase, $\varphi,$ of the periods considered on both timescales (ST, LT). This is important, because it allows to inspect temporal delays between the variables on the two timescales, and uncovers possible synchronization that is otherwise masked by low-periodicity phenomena or lost by the lack of angular phase information (when reconstructing the signals only based on their spectral amplitude). Obviously, the mere existence of common periods (on the two timescales) between the SSA, PG, and NC does not prove the existence of any relation between the solar cycle activity and Earth’s atmosphere. However, if a clear similarity is evident between the trends in the three studied parameters, a possible link between these parameters might be hypothesized. To uncover such a link, we used the PSR.

First, to compare between the three variables studied here, we performed the following normalization transformation:

1 $$X(t) = \frac{{x(t) - \bar{x}}}{{\sigma_{x} }}.$$

where $\bar{x}$ and $\sigma_{x}$ are the mean and the standard deviation of *x*, respectively. Similar transformations were performed for $v(t) \equiv \dot{x}(t)$ and *V*(*t*). These transformations normalize the time series and make them dimensionless; this procedure is usually referred to as data standardization. In addition, time derivatives were discretized, as follows: $\dot{x}(t) \cong \Delta x(t)/\Delta t$ and $\Delta x(t) = x(t) - x(t - \Delta t)$, with Δ*t* = 1 day.

**Fig. 3:**
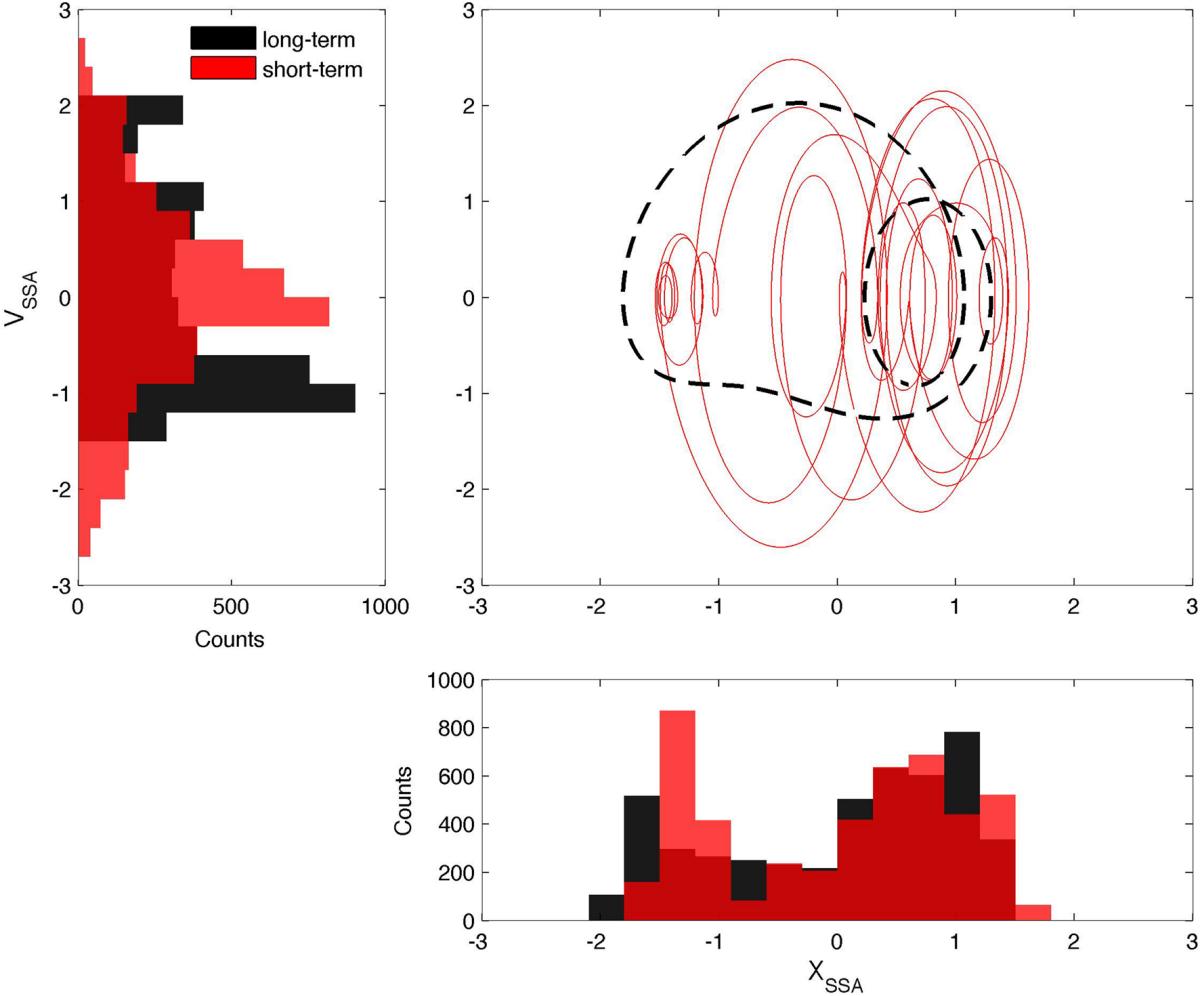
Phase space representation of the sunspot area in the form of *X*_SSA_ versus *V*_SSA_ (c*entral panel*). The *solid red line* represents the short-term case, while the *dashed black line* stands for the long-term case. *Left panel* histogram representation of the *V*_SSA_. *Bottom panel* histogram representation of the *X*_SSA_

**Fig. 4:**
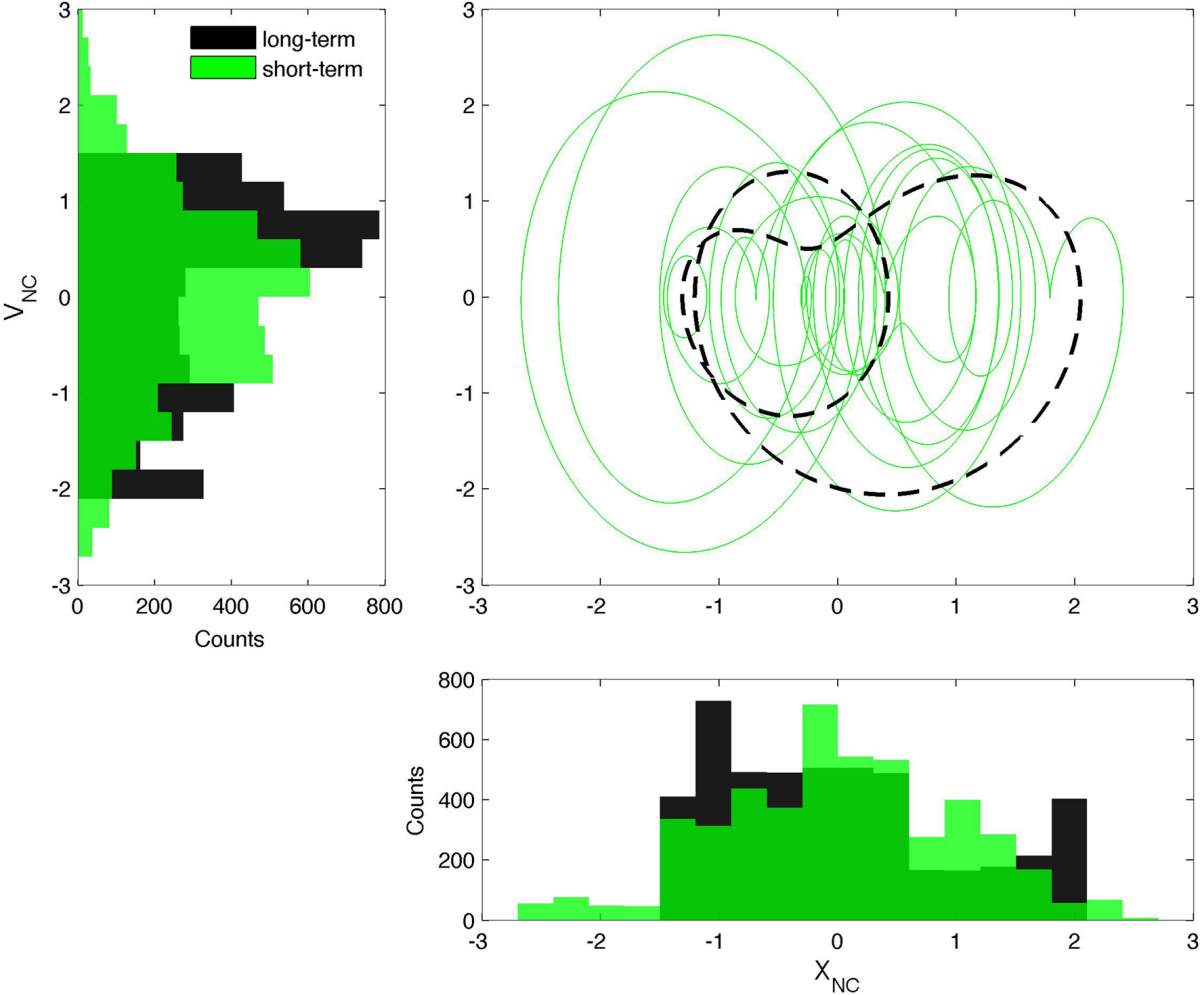
Phase space representation of the neutron monitor count rate in the form of *X*_NC_ versus *V*_NC_ (c*entral panel*). The *solid green line* represents the short-term case, while the *dashed black line* stands for the long-term case. *Left panel* histogram representation of the *V*_NC_. *Bottom panel* histogram representation of the *X*_NC_

**Fig. 5:**
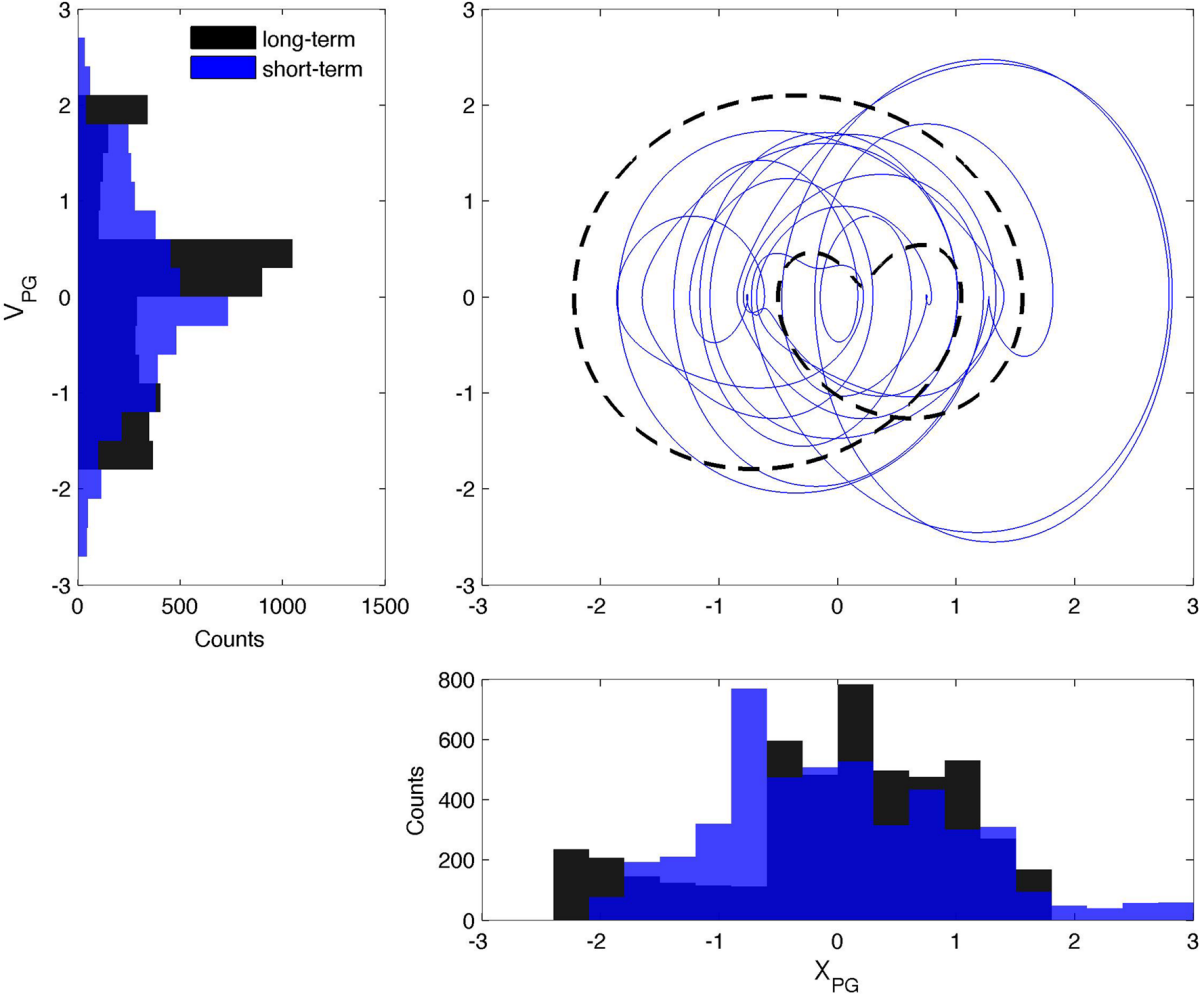
Phase space representation of the potential gradient in the form of *X*_PG_ versus *V*_PG_ (c*entral panel*). The *solid blue line* represents the short-term case, while the *dashed black line* stands for the long-term case. *Left panel* histogram representation of the *V*_PG_. *Bottom panel* histogram representation of the *X*_PG_

**Fig. 6:**
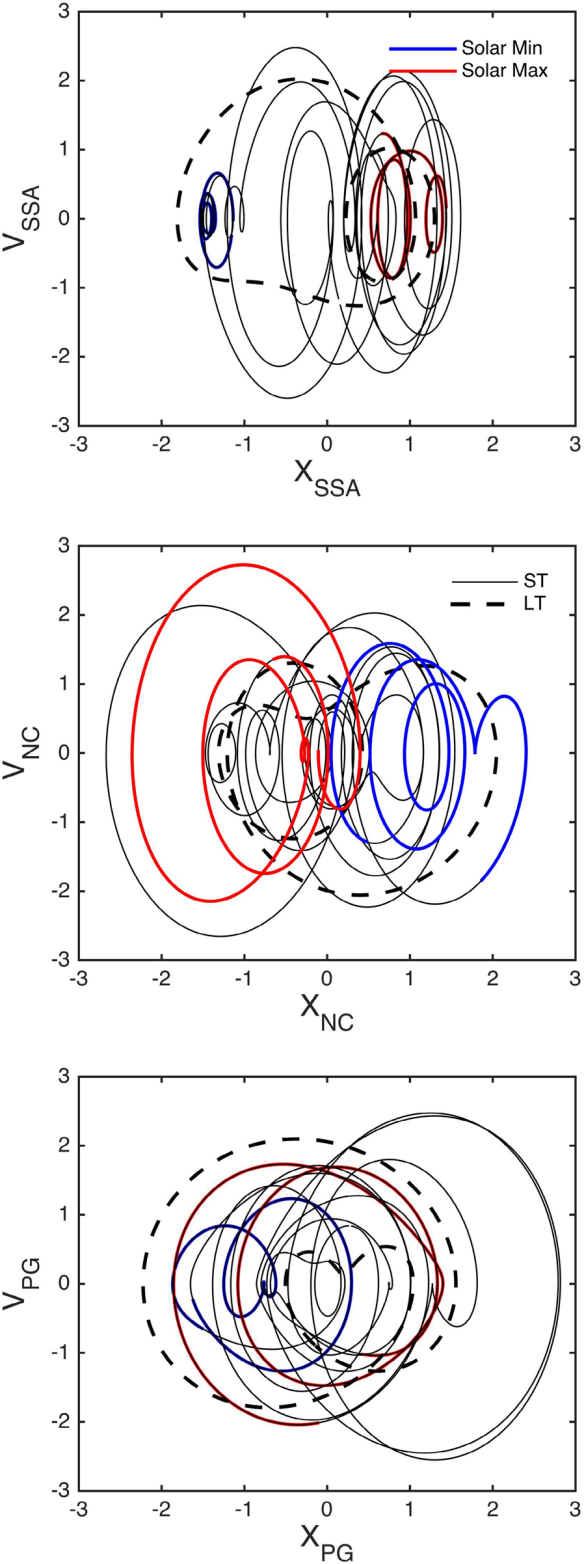
Phase space representation highlighting the short-term behavior at the solar maximum (*red curve*) and solar minimum (*blue curve*), against the long-term behavior (*dashed curve*), for: the sunspot area (*upper panel*), the neutron monitor count rate (*middle panel*), and the potential gradient (*lower panel*)

Second, in Figs. 3, 4, and 5, the signals are shown in their PSRs, with solid colored lines for ST and dashed black lines for LT. In terms of chaos theory, these figures reveal the existence of attractors, but a detailed discussion of this aspect is out of the scope of the present work. Similar topological features are seen in the three representations of both the ST and LT signals. Analysis of the LT signals suggests the existence of a link between the three variables and the solar cycle activity; this link will be explored in depth in what follows. The ST signals exhibit Gaussian distributions for both *X* and *V*, indicative of the influence of the ST variability. An exception is made for *X*_SSA_, which exhibits a bimodal distribution, where the two peaks correspond to the solar maximum and minimum, respectively. Similar bimodal distributions characterize all of the LT signals. This confirms that the LT signals are related to the solar cycle activity, with the PSR composed of two closed curves: a small curve (corresponding to the solar maximum) inside a larger curve (matching the solar minimum). The LT curve of the NC is a reflection of the SSA and PG ones, in relation to the diagonal of the *xx* and *yy* axis, revealing a topological anti-correlation. Further analysis of the ST representation of the SSA, as shown in Fig. 6, revealed that the ST curves for the 1979 solar maximum (in red and related to the first shaded area in Fig. 2) tend to be concentrated inside the LT curve corresponding to a similar solar cycle activity, and such observation is also valid for the 1986 solar minimum (in blue and related to the second shaded area in Fig. 2). This highlights a modulation of the ST signal by the LT solar cycle activity in the case of the SSA, which is compatible with the bimodal distribution of *X*_SSA_. Such a trend is weaker for the NC and even weaker for the PG, where the ST curves often overlap, but a distinction can still be made, justifying a possible LT modulation of the ST signal tentatively attributed to the solar cycle activity. In fact, if the LT component is removed from the ST signal, the curves in the PSR converge approximately to a single circle, as expected for a simple harmonic oscillator.

**Fig. 7:**
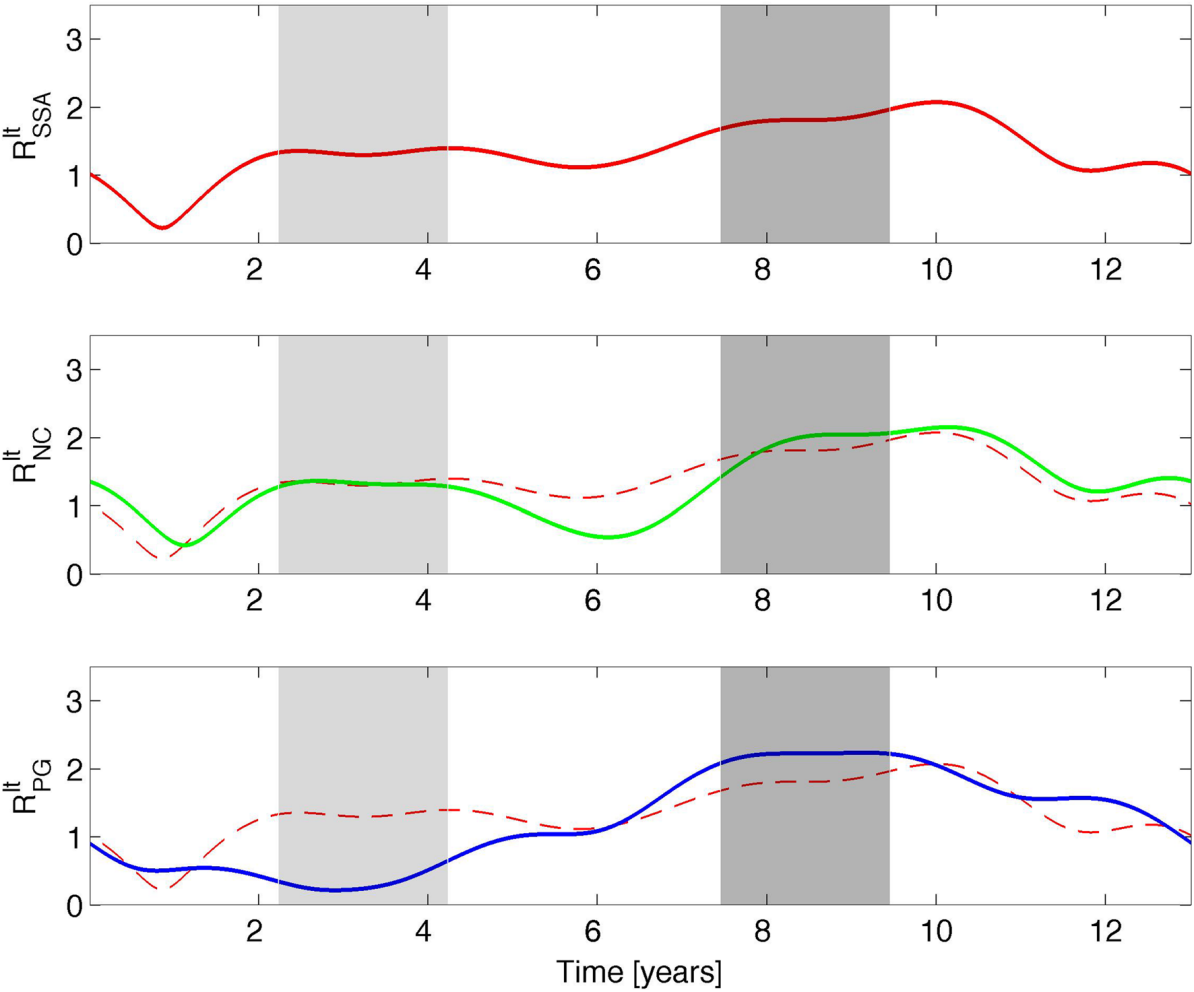
Long-term (preserving the periods ~13.00, ~6.50, and ~4.34 years) phase space radius, *R*, for: the sunspot area (*upper panel*), the neutron monitor count rate (*middle panel*), and the potential gradient (*lower panel*). The *first gray*-*shaded area* corresponds to the 1979 solar maximum, and the *second gray*-*shaded area* relates to the 1986 solar minimum

**Fig. 8:**
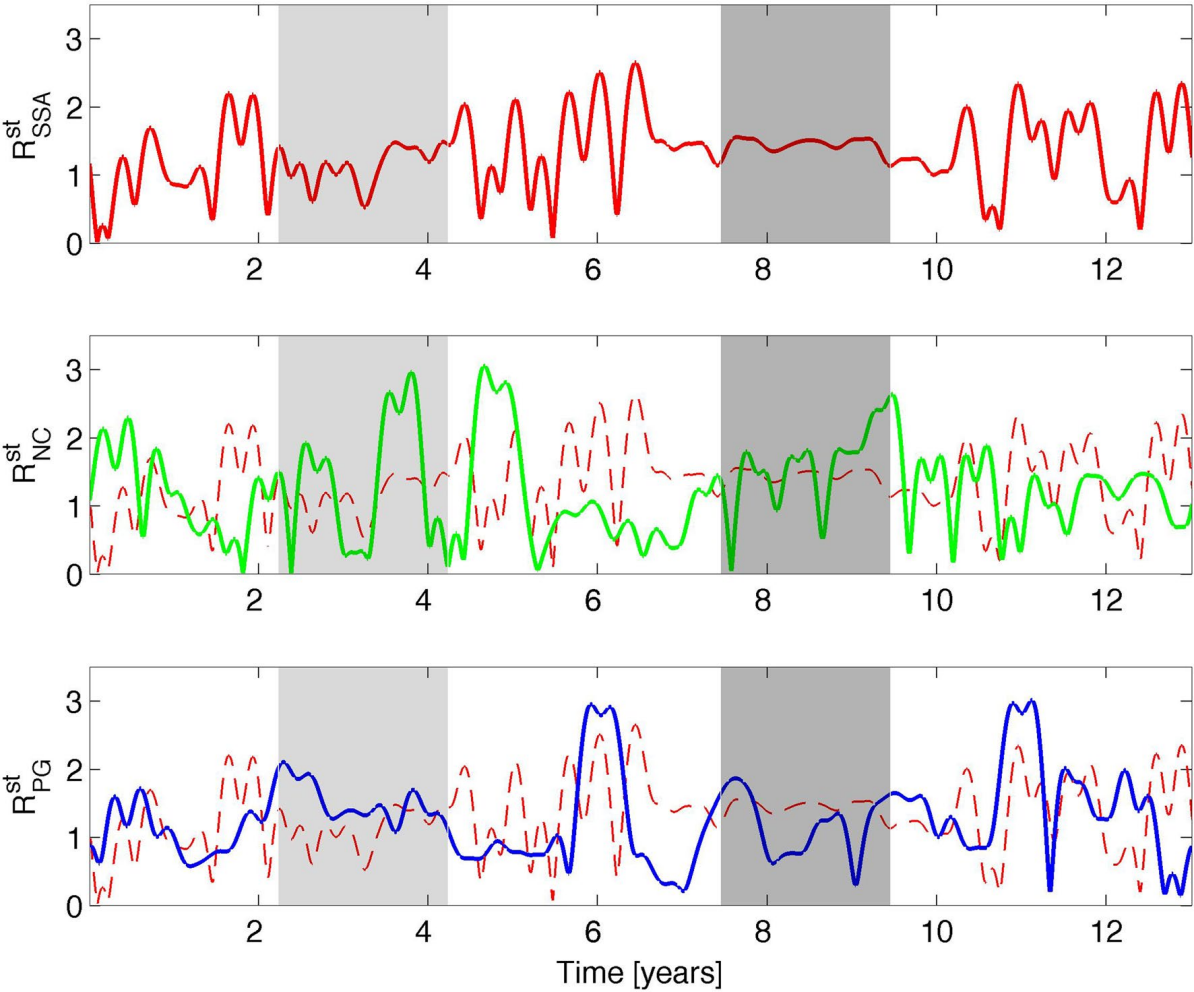
Short-term (periods ranging from ~0.50 to ~13.00 years) phase space radius, *R*_st_, for: the sunspot area (*upper panel*), the neutron monitor count rate (*middle panel*), and the potential gradient (*lower panel*). The *first gray*-*shaded* area corresponds to the 1979 solar maximum, and the *second gray*-*shaded* area relates to the 1986 solar minimum

To pursue the existence of nonlinear relations among the three variables on both timescales, we calculated the dimensionless phase space radius, *R*, defined as:

2 $$R = \sqrt {X^{2} + V^{2} } ,$$

where *X* and *V* were calculated according to Eq. (1) for the SSA, NC, and PG. This dimensionless parameter besides its geometrical meaning represents, by analogy with the simple harmonic oscillator, the energy balance between the potential energy $( \propto X^{2} )$ and the kinetic energy $( \propto V^{2} )$ of the nonlinear oscillator. Figure 7 shows the LT behavior of *R* for the SSA, NC, and PG, while Fig. 8 shows the ST behavior of *R*_st_. To compare the SSA behavior with those for the NC and PG, the *R* and *R*_st_ results for the SSA were replotted as dashed red lines in both the NC and PG plots. The correlations between the values of *X*, *V*, and *R* for the three variables are listed in Table 1, and the results for *X*_st_, *V*_st_, and *R*_st_ are listed in Table 2.

**Table 1:** Correlation coefficients, *r*, and the respective *p* values for the long-term (preserving only higher periods, ~13.00, ~6.50, and ~4.34 years—lt) relation between: the sunspot area and neutron monitor count rate (SSA–NC), the sunspot area and potential gradient (SSA–PG), and the neutron monitor count rate and potential gradient (NC–PG)

	SSA–NC	SSA–PG	NC–PG
	r*	r*	r*
*X*	−0.8760	0.7042	−0.7931
*V*	−0.8882	0.7030	−0.6989
*R*	0.8271	0.6978	0.6241

* All of the correlation coefficients have *p* values <0.0001

**Table 2:** Correlation coefficients, *r*, and the respective *p* values for the short-term (periodicities ranging from ~0.50 to ~13.00 years—st) relation between: the sunspot area and neutron monitor count rate (SSA–NC), the sunspot area and potential gradient (SSA–PG), and the neutron monitor count rate and potential gradient (NC–PG)

	SSA–NC	SSA–PG	NC–PG
	*r* (*p* value)	*r* (*p* value)	*r* (*p* value)
*X*_st_	−0.6058 (<0.0001)	0.3922 (<0.0001)	−0.2133 (<0.0001)
*V*_st_	−0.1873 (<0.0001)	0.1116 (<0.0001)	−0.2212 (<0.0001)
*R*_st_	−0.1555 (<0.0001)	0.040 (~0.006)	0.0 (~0.3)

On the LT timescale, a clear agreement appears to be present for the different correlations found for *X* and *V*: Significant anti-correlations were observed between the SSA and NC and between the NC and PG; a significant correlation was found between the SSA and PG. If a causality relation exists between the SSA and PG, mediated by the GCRs (here represented by the NC), it is expected that the Pearson correlation coefficient, *r*, will obey *r*_SSA–PG_ ~ *r*_SSA–NC_ × *r*_NC–PG_. This relation was found to be valid for *X*, but not for *V*, for which it was found that |*r*|_SSA–PG_ ~ |*r*|_NC–PG_ and |*r*|_SSA–NC_ ~ 0.89. The variable *V* represents the variation rate of *X* and although the deviation from the causality relation is relatively slight, it might indicate an atmospheric mechanism that decouples the LT variation in the NC from the variation in the PG, reinforced by the fact that for *X*, the anti-correlation between the two variables, *r*_NC–PG_ ~ −0.79, decreases to *r*_NC–PG_ ~ −0.70 for *V*; both coefficients have *p* values <0.0001. This further shows the importance of inspecting both *X* and *V* when studying nonlinear systems. Analyzing now *R*, Fig. 7 shows that the LT behavior of the NC and PG follows the one of the SSA, although the NC demonstrates a better agreement. In fact, the PG behavior seems to deviate from the SSA during the 1979 solar maximum (first gray-shaded area in Fig. 7). Such a deviation of the PG in relation to the SSA is reinforced by the correlation coefficients. When comparing *R* for the SSA and NC, a value of *r*_SSA–NC_ ~ 0.83 was obtained, which is higher than the value obtained for the correlation between the SSA and PG, *r*_SSA–PG_ ~ 0.70; both coefficients have *p* values <0.0001. Three conclusions are made by analyzing the results in Table 1:

1. The SSA–PG correlation coefficient is nearly the same for *X*, *V*, and *R*, revealing a robust relation between the two variables.
2. The negative correlation between the SSA and NC when considering *X* and *V* changes to the positive correlation when using *R*, while its absolute value remains high; this is expected because *R* describes the nonlinear nature of the systems, and in fact, both variables exhibit a similar nonlinear behavior.
3. An analogous change from correlation to anti-correlation takes place for the NC–PG relation, but (in terms of the absolute value) the correlation coefficient decreases from *X* to *V* and from *V* to *R*; this reveals the influence of an LT decoupling mechanism (as discussed above).

Finally, the correlation coefficients of *R* for the SSA, NC, and PG reveal a different causality relation when compared with those of *X*, which are defined by: *r*_NC–PG_ ~ *r*_SSA–NC_ × *r*_SSA–PG_. Estimating the correlation coefficients using this formula yields *r*_NC–PG_ ~ 0.58; this value differs by only 7.5 % from the determined one *r*_NC–PG_ ~ 0.62, as listed in Table 1. This finding (the most sounding of this part of the analysis) reveals a significant LT synchronization between the SSA and NC and PG, respectively, and such a synchronization underlies the NC–PG relation. This constitutes evidence of a synchronization mechanism between two parameters from the Earth’s atmosphere, NC and PG, and solar cycle activity. Other Earth variables (e.g., solar radiation) may be subjected to a similar analysis in future works.

The next step in the analysis is to verify whether this synchronization still holds on the ST timescale. It is expected that the high-frequency variability of the ST signals would mask the LT relation and synchronization would not be visible anymore. According to this, Table 2 shows an overall reduction in the absolute values of the correlation coefficients. This reduction in the correlation is particularly visible for *R*_st_ which, as shown in Fig. 8, shows almost no agreement between the SSA and NC and PG. Indeed, Table 2 reveals no correlations for this parameter in the SSA–PG and NC–PG cases, and a weak anti-correlation is found in the SSA–NC case; this suggests that the SSA and NC are more significantly related one to another than to PG. Naturally, the PG is more affected by an ST atmospheric variation (e.g., turbulence) and this explains why an ST relation is found only in the SSA–NC case. Anyway, the correlation coefficient is too weak to validate any actual ST relation between the SSA and NC, based only on *R*_st_. Note that in the case of *X*_st_, the correlation coefficients in Table 2 suggest significant anti-correlations between the SSA and NC and NC and PG, and a correlation between the SSA and PG (similar to the LT results). The absolute value of the correlation coefficient of *X*_st_ in the SSA–NC case was |*r*|_SSA–NC_ ~ 0.61; in the SSA–PG case, this value decreased to |*r*|_SSA–PG_ ~ 0.39, and in the case of NC–PG it was |*r*|_NC–PG_ ~ 0.21; the three coefficients had *p* values <0.0001. Even so, a causality rule of the type *r*_NC–PG_ ~ *r*_SSA–NC_ × *r*_SSA–PG_ was still valid. This formula yielded *r*_NC–PG_ ~ −0.24; this value differs by ~12 % from the determined one (*r*_NC–PG_ ~ −0.21, Table 2). For this reason, synchronization on the ST timescale is still hypothesized, although it is likely to be less significant than the one found on the LT timescale. Finally, in relation to *V*_st_ a noteworthy reduction was found for the absolute values of correlation coefficients in the SSA–NC and SSA–PG cases in relation to *X*_st_, but the anti-correlations found in the NC–PG case for both *V*_st_ and *X*_st_ were similar. This last observation could be attributed to the high variability imposed by the atmosphere on these two variables, and it is manifested as a vanishing correlation when considering *R*_st_, resembling a destructive interference between *X*_st_ and *V*_st_ that is typical of noise.

**Fig. 9:**
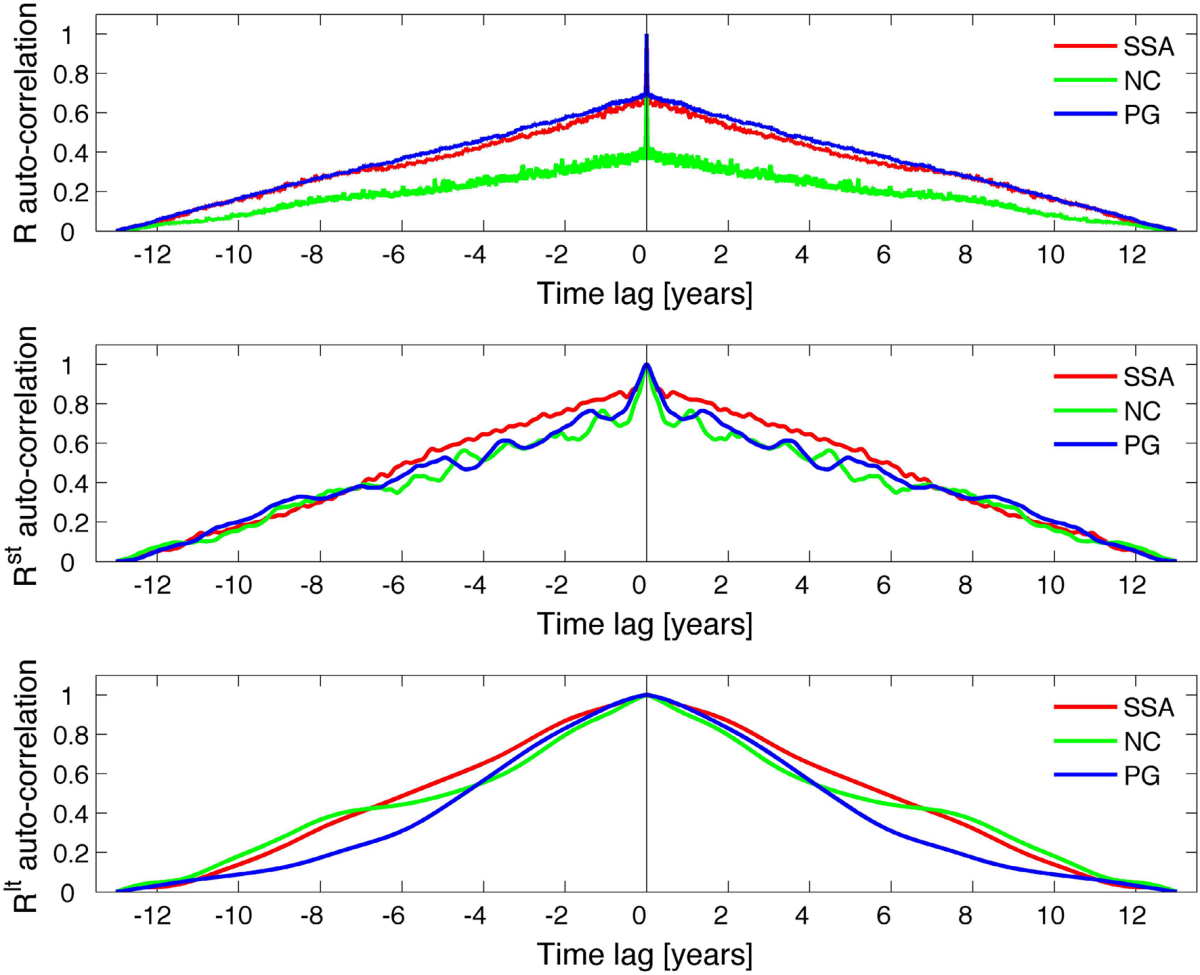
Autocorrelation functions for *R*, calculated on the raw data (*upper panel*), and with short-term (*middle panel*) and long-term (*lower panel*) smoothings

Finally, we comment on the validity of the presented correlation values. Considering heavily smoothed time series raises the issue of autocorrelations, because time series autocorrelation substantially increases the correlation between different time series. This leads to an overestimation of a true correlation; considerable attention was given to this in the literature (e.g., Dunn 2005). For the sake of clarity, the autocorrelations of *R* were calculated on the raw data and using the ST/LT smoothing, and the results are shown in Fig. 9. It is clearly seen that similar autocorrelation functions are found on the three timescales; similar results hold for *X* and *V*. This constitutes a first indication that autocorrelation does not affect previously found correlations. Resampling the smoothed time series using lower sampling rates and calculating correlations for the resampled time series constitute a second test. As an example, *R* was considered for the following four cases: (1) one sample every year, (2) one sample every 2 years, (3) one sample every 3 years, and (4) one sample every 4 years. The results are shown in Table 3 and indicate no sizeable difference between the correlation coefficients in the four resampling cases, compared with the correlations listed in Table 1. This proves that the obtained correlations are statistically significant. Naturally, the *p* values change as a consequence of the reduction in the number of data samples when decreasing the sampling rate from one sample every year to one sample every 4 years.

**Table 3:** Correlation coefficients for *R*_lt_, for different resampling times

Resampling time (years)	SSA–NC	SSA–PG	NC–PG
	*r* (*p* value)	*r* (*p* value)	*r* (*p* value)
1	0.8260 (~0.0002)	0.693 (~0.006)	0.61 (~0.02)
2	0.83 (~0.02)	0.7 (~0.1)	0.6 (~0.1)
3	0.7 (~0.1)	0.6 (~0.3)	0.5 (~0.4)
4	0.9 (~0.1)	0.6 (~0.4)	0.7 (~0.3)

## Conclusions

Relating the NC and PG with SSA using phase space techniques produced two main findings:

1. Similar short-term topological features of the PSR of the three variables, indicating similar nonlinear behaviors.
2. A long-term phase space radius synchronization of the solar cycle activity with the NC and PG, confirmed by absolute correlation coefficients above ~0.8.

These findings serve as a motivation to use low-order dynamic models that can be reduced to equations of known nonlinear oscillators, as a mathematical description of these signals. In properly determined models, the constants in the corresponding equations can provide deep insights into the physical mechanisms underlying short-term and long-term relations found in this work. In this perspective, the present work is likely to be a promising contribution to space–earth weather research. The methodology proposed here can be used for obtaining the relations between other atmospheric parameters (e.g., solar radiation) and solar cycle activity.

## Footnotes

1 The convention is that the potential gradient is PG = d*V*_I_/d*z*, where *V*_I_ is the potential with respect to Earth’s surface (where *V*_I_ = 0) and *z* is the vertical coordinate. Using this convention, the PG is positive for fair-weather days and related to the vertical component of the atmospheric electric field *E*_z_ by *E*_z_ = −PG.

2 NC results from secondary particles produced by GCRs in Earth’s atmosphere.
